# Fast Nondestructive Detection Technology and Equipment for Food Quality and Safety

**DOI:** 10.3390/foods12203744

**Published:** 2023-10-12

**Authors:** Zhiming Guo, Heera Jayan

**Affiliations:** 1 School of Food and Biological Engineering, Jiangsu University, Zhenjiang 212013, China; 2 International Joint Research Laboratory of Intelligent Agriculture and Agri-Products Processing, Jiangsu University, Zhenjiang 212013, China; 3 China Light Industry Key Laboratory of Food Intelligent Detection & Processing, Jiangsu University, Zhenjiang 212013, China

Fast nondestructive detection technology in food quality and safety evaluation is a powerful support tool that fosters informatization and intelligence in the food industry, characterized by its rapid processing, convenient operation, and seamless online inspection. Over the past two decades, these technologies have found numerous successful applications in the field of food and agricultural product detection and processing. Owing to improvements in the development of photoelectric sensors and the ongoing progress in artificial intelligence and software algorithms, fast nondestructive detection technologies provide significantly enhanced accuracy, reliability, and stability, revolutionizing their role in food quality and safety detection and processing. Their seamless integration with the Internet of Things (IoT) and intelligent manufacturing is promoting a new wave of innovation in the food industry. The application of new sensing technology and equipment in the fast, nondestructive detection of food has always been at the forefront of scientific and technological research. The schematic diagram of the advance in research progress is shown in Figure 1. This Special Issue is dedicated to highlighting the latest research progress and jointly discussing the future directions of research and development in the field.

Raman spectroscopy is a fast and sensitive tool that has established itself as a valuable technique that has demonstrated successful applications in ensuring food safety and quality. Yin et al. [1] employed bimetallic core–shell nanoparticles and a specific redox reaction of carbimazole and chromium iron for the surface-enhanced Raman spectroscopy (SERS) detection of hexavalent chromium in tea. The developed techniques demonstrated excellent sensitivity, emphasizing the significant potential of rapid, non-destructive, and sensitive SERS detection in the field of food safety and quality analysis. Qiu et al. [2] developed an SERS-based method for the detection of polycyclic aromatic hydrocarbon (PAH) residues on the surface of fruits and vegetables. A flexible substrate (β-CD@AuNP/PTFE) was employed for enhancing the signals along with lightweight deep learning networks for data analysis. In addition, Zhang et al. [3] utilized a microfluidic chip for the capture of crop airborne disease spores for further detection using Raman spectroscopy. The use of support vector machine (SVM) and back-propagation artificial neural network (BPANN) ensured high accuracy in detection. Thus, the integration of deep learning in Raman spectroscopic data facilitates automated feature extraction, accommodates complex data relationships, and achieves high accuracy levels, thereby enabling its effective application in the field of food safety. Interestingly, Sun et al. [4] focused on theoretically calculating Raman spectra for five commonly used plasticizers, known as phthalic acid esters (PAEs). The density functional theory (DFT) calculations showed in the research have the potential to contribute to the development of Raman spectroscopic methods for the rapid detection of PAEs in the future, a crucial step in assessing their potential health risks. These innovative applications demonstrate the effectiveness of Raman spectroscopy in detecting contaminants and analyzing the quality of food products. Theoretical advancements in Raman spectral calculation help to gain insights into molecular structure, composition, and their interaction, which eventually has the potential to improve the accuracy and sensitivity of Raman-based analysis.

Further, Sun et al. [5] employed visible/near-infrared (Vis/NIR) spectroscopy to detect the soluble solid content in fresh jujubes along with a least square support vector machine to develop a model. The proposed method yielded highly accurate prediction results, effectively tackling the demand for quality analysis of jujubes in the open fields. In addition, Jiang et al. [6] developed a calibration method for NIR spectroscopy to enhance the accuracy of the model for detecting the soluble solid content in apples of different sizes. The results hold high significance in advancing the development of dependable models for predicting the SSC in diverse fruits. While establishing a Vis/NIR spectroscopy detecting method for the stone cell content of Korla fragrant pears, Wang et al. [7] showed that the standardized normal variate (SNV) pre-processed successive projective algorithm–support vector regression (SPA-SVR) model effectively meets the requirements for intelligent evaluation, achieving high correlation coefficients for both calibration and validation sets. The effectiveness of Vis/NIR spectroscopy in food quality analysis was further proved by Wang et al. [8], where Vis/NIR spectroscopy was successfully employed to predict the anthocyanin content in purple Chinese cabbage with high accuracy. On the other hand, Migues et al. [9] developed a method for predicting the acceptability of Mandarin fruit based on the sugar and citric acid levels extracted from the NMR spectroscopic data. The study proved that the chemometric-based models facilitate data-driven decisions to optimize food quality, ensuring that the product meets consumer demands and regulatory standards. In addition, He et al. [10] evaluated the impact of ^60^Co irradiation on turmeric essential oil composition using gas chromatography–ion mobility spectrometry (GC–IMS). The findings demonstrated that, even though compound composition remained constant, the peak intensities were altered, supporting a 5 kGy/min irradiation dose for preserving essential oil quality. The studies have effectively demonstrated that the integration of spectroscopic techniques along with advanced data analysis is a promising choice that cements the advancement in food safety and quality analysis. These approaches offer rapid and accurate predictions of key quality attributes in various food products.

The ability of machine learning to extract valuable information from high-dimensional spectral data was utilized to enhance the effectiveness and efficiency of hyperspectral imaging in analyzing the safety and quality of food products. Xu et al. [11] explored the relationship between water distribution and quality indicators in shrimp during hot air drying using hyperspectral imaging. The study revealed a positive association between shrimp moisture content and bound water, immobilized water, and free water. Conversely, attributes including hardness, stickiness, and chewiness showed negative correlations with bound water and free water. Likewise, Cao et al. [12] developed a rapid approach for assessing the texture profile analysis of common carp fillets, leveraging hyperspectral imaging and machine learning algorithms. The proposed method accelerated the assessment process and maintained the integrity of the product, making it a valuable alternative to traditional texture analysis methods. Additionally, Wang et al. [13] and Xu et al. [14] both employed hyperspectral imaging to assess the quality of the safety of maize seeds. However, Wang et al. [13] developed a method to detect mold growth in maize kernels by applying categorical analysis and data fusion to hyperspectral data. In contrast, Xu et al. [14] developed a method to identify defective maize seeds by employing deep learning, particularly convolutional neural networks to hyperspectral images. Further, Zhang et al. [15] combined internal and external leaf features obtained from both near-infrared hyperspectral imaging and THz time-domain spectroscopy to assess the different grades of tomato leaf mildew infestation. The fusion of these sources of information allows for a high degree of accuracy in detection, preventing misdiagnosis associated with traditional disease detection methods. Hyperspectral imaging has brought about a transformative shift in food quality and safety assessment, providing an in-depth analysis of food products. The wide range of applications of hyperspectral imaging from understanding water distribution and assessing texture to detecting mold growth and defects emphasizes its versatility and reliability.

IoT plays a significant role in food safety and quality assurance by providing real-time monitoring, data accuracy, complete traceability, and early warning systems throughout the food supply chain. Yin et al. [16] developed a spoilage monitoring and early warning system based on the volatile component production during apple spoilage. The combination of a sensor prototype and multi-factor fusion early warning model provided the real-time evaluation of food spoilage. Thus, the development of novel sensors that have the capability to collect data from the environment is an integral part of the IoT to ensure miniaturization and energy efficiency. In this regard, a chemiresistive ethylene sensor, employing rGO/WSe2/Pd heterojunctions, has been developed for room-temperature (RT) ethylene detection. This sensor offers a practical solution to monitor ethylene concentration, improving fruit and vegetable quality control during transportation and reducing losses [17]. Similarly, Zhang et al. [18] developed a bi-layer containing an anthocyanin-loaded liposome that has the capability to indicate the freshness of shrimp products through visual color changes by monitoring the pH of the surrounding medium. These studies have great significance as they focus on sensor development, which is a key aspect of smart and intelligent agriculture.

Artificial intelligence and machine learning can provide a conceptual tool to transform food safety and quality data management, facilitating the early detection and prevention of food safety issues. The synergy between deep learning and image processing was harnessed by Liang et al. [19] to develop a real-time grading system for defective apples using an RGB camera machine vision system and a combination of semantic segmentation and a pruned YOLO V4 network. This approach ensured a high detection accuracy (92.42%) without compromising computational efficiency. In addition, Zhou et al. [20] found that the light penetration depth in apple tissues was around 2.2 mm when spatial frequency domain imaging (SFDI) was used to detect early stage bruises in apple tissue. These works proved the effectiveness of deep learning architecture in detecting early stage defects in thin-skinned fruits. Moreover, Chen et al. [21] focused on creating a methodology for assessing the degree of milling (DOM) in rice with digital image processing technology and deep learning. The research introduced an enhanced model that combines multi-scale information through the integration of the Inception-v3 structure and the residual network (ResNet) model, using the Bayesian optimization algorithm which achieved superior results. The method achieved an average detection accuracy of 96.9%. Similarly, Yu et al. [22] developed a model using YOLOv5 to identify small impurities in walnut kernels that showed a detection accuracy of 88.9%. The model achieved a faster detection time for single images using an improved YOLOv5 model. By replacing conventional Conv with Ghostconv, the detection time was reduced from 65.25 ms to 45.38 ms, ensuring the real-time detection of walnut impurities while maintaining detection performance.

Ensuring the safety and quality of food products is of paramount importance in the food supply chain. From production to distribution, rigorous food safety and quality inspections have to be conducted at every stage involving the monitoring and control of food processing methods, preventing contamination and maintaining the highest standard. Ongoing research and development efforts focus on creating non-destructive technologies and cutting-edge equipment to attain this goal. These innovations understandably play a crucial role in ensuring the safety and quality of food products without compromising the integrity and promoting public safety and confidence in the food supply chain. The application of artificial intelligence, big data, and the IoT has led to a transformative era for the food industry. This implementation has ushered in an improvement in quality and an increase in efficiency across the entirety of the food production and distribution chain. Predictive analytics powered by artificial intelligence help to optimize production and minimize postharvest loss, while IoT-associated sensors provide real-time data on various environmental factors, ensuring food safety and quality. Big data analytics provides insight into consumer preferences and market trends, leading to more informed decision making. These digital transformations promote the transformation of and upgrade the food industry, making it more sustainable, innovative, and responsive to the evolving needs of consumers.

In summary, this Special Issue explores a wide range of innovative research at the intersection of technology development, artificial intelligence, and IoT. From sensor development and emerging techniques to machine learning and chemometric analysis, the studies included in this Special Issue showcase the incredible progress in safety and quality analysis in the food industry. Moving forward, these advances hold great importance in revolutionizing, early detection, quality assessment, and safety evaluation, ultimately benefiting both consumers and the food industry.

## Figures and Tables

**Figure 1:**
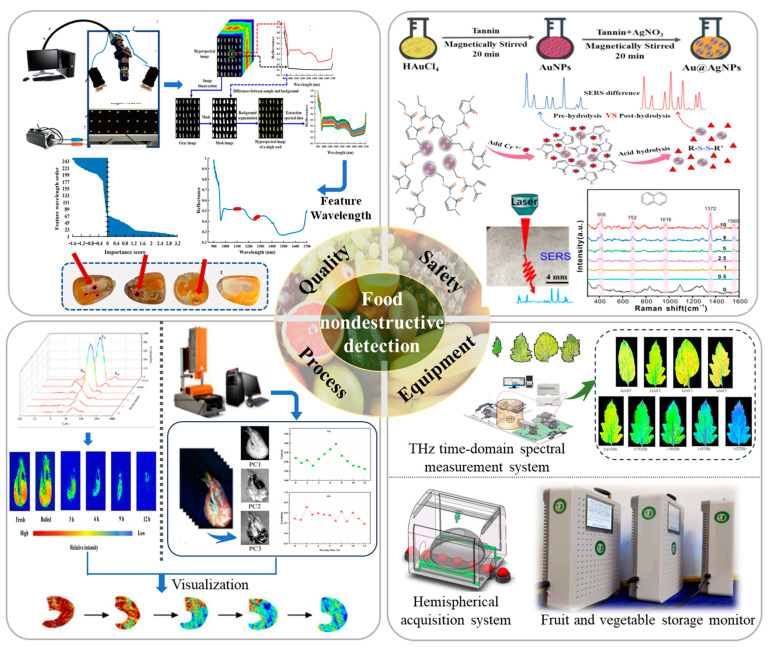
Schematic diagram of the advance research progress of fast nondestructive detection technology and equipment for food quality and safety.

## Data Availability

Referred datasets link at https://www.mdpi.com/journal/foods/special_issues/Fast_Non_Destructive_Detection_Technology_Equipment_Food_Quality_Safety (accessed on 3 October 2023).
